# Differential Production of Midkine and Pleiotrophin by Innate APCs upon Stimulation through Nucleic Acid-Sensing TLRs

**DOI:** 10.1155/2023/7944102

**Published:** 2023-10-09

**Authors:** Elias A. Said, Sumaya Al-Dughaishi, Wadha Al-Hatmi, Iman Al-Reesi, Mohammed S. Al-Balushi, Atika Al-Bimani, Juma Z. Al-Busaidi, Marwa Al-Riyami, Murtadha Al-Khabori, Salam Al-Kindi, Francesco A. Procopio, Shadia Al-Sinawi, Aliyaa Al-Ansari, Crystal Y. Koh, Khalid Al-Naamani, Ali A. Al-Jabri

**Affiliations:** ^1^Department of Microbiology and Immunology, College of Medicine and Health Sciences, Sultan Qaboos University, Muscat, Oman; ^2^Department of Pathology, College of Medicine and Health Sciences, Sultan Qaboos University, Muscat, Oman; ^3^Department of Hematology, College of Medicine and Health Sciences, Sultan Qaboos University, Muscat, Oman; ^4^Service of Immunology and Allergy, Lausanne University Hospital (CHUV), Lausanne, Switzerland; ^5^Department of Biology, College of Science, Sultan Qaboos University, Muscat, Oman; ^6^Department of Medicine, Armed Force Hospital, Muscat, Oman

## Abstract

Midkine (MK) and pleiotrophin (PTN) belong to the same family of cytokines. They have similar sequences and functions. Both have important roles in cellular proliferation, tumors, and diseases. They regulate and are expressed by some immune cells. We have recently demonstrated MK production by some human innate antigen-presenting cells (iAPCs), i.e., monocyte-derived dendritic cells (MDDCs) and macrophages stimulated through Toll-like receptor (TLR)-4, and plasmacytoid dendritic cells (pDCs) stimulated through TLR 7. While PTN production was only documented in tissue macrophages. TLRs 3, 7, 8, and 9 are nucleic acid sensing (NAS) TLRs that detect nucleic acids from cell damage and infection and induce iAPC responses. We investigated whether NAS TLRs can induce MK and PTN production by human iAPCs, namely monocytes, macrophages, MDDCs, myeloid dendritic cells (mDCs), and pDCs. Our results demonstrated for the first time that PTN is produced by all iAPCs upon TLR triggering (*p* < 0.01). IAPCs produced more PTN than MK (*p* < 0.01). NAS TLRs and iAPCs had differential abilities to induce the production of MK, which was induced in monocytes and pDCs by all NAS TLRs (*p* < 0.05) and in MDDCs by TLRs 7/8 (*p* < 0.05). TLR4 induced a stronger MK production than NAS TLRs (*p* ≤ 0.05). Monocytes produced higher levels of PTN after differentiation to macrophages and MDDCs (*p* < 0.05). The production of MK and PTN differs among iAPCs, with a higher production of PTN and a selective induction of MK production by NAS TLR. This highlights the potentially important role of iAPCs in angiogenesis, tumors, infections, and autoimmunity through the differential production of MK and PTN upon TLR triggering.

## 1. Introduction

Midkine (MK) and pleiotrophin (PTN) are the members of the MK/PTN family of heparin-binding cytokines [1]. MK is a 13 kDa protein that has 143 amino acids, and the *MDK* gene is located on the 11q11.2 chromosome in human [2], while PTN is an 18 kDa protein that has 168 amino acids [3], and the *PTN* gene is located on the 7q33 chromosome in human [4]. MK and PTN share around 50% of their amino acid sequences [3], and this includes the basic amino acids that are crucial for their function and structure. Both of their sequences are highly conserved among species in mammals [1]. They demonstrate similarities in their conserved N- and C-domains that have disulfide bonds and are linked to each other by a hinge [5]. The C-domain of both MK and PTN is essential for their biological activities [5].

Moreover, MK and PTN have similarities in their functions. Their expression is increased in many types of cancers, and they have an important role in tumor cell proliferation and metastasis [2]. They enhance the proliferation of different types of cells, e.g., epithelial cells, endothelial cells, fibroblasts, and tumor cell lines. In addition, both MK and PTN can affect lymphocyte, macrophage, and neutrophil activities [5]. Importantly, these two cytokines have a major implication in the development of embryos, especially in the development of the nervous system and cell differentiation. In the mouse model, the deficiency in MK or PTN genes is associated with some behavioral abnormalities, e.g., a working memory deficiency or higher levels of anxiety, and the absence of the two genes simultaneously results in more severe effects [6]. MK and PTN are also implicated in stem cell renewal and cell survival [7, 8].

MK and PTN were also shown to be associated with different diseases, including cancers [2, 9] and autoimmune diseases such as rheumatoid arthritis (RA) [10, 11], systemic lupus erythematosus (SLE) [3], and multiple sclerosis (MS) [12, 13]. We and others have shown that MK and PTN binding to their receptors can inhibit HIV infection by interfering with the capacity of the virus to bind to target cells [14–16]. MK and PTN share many of their receptors. Both cytokines bind to Syndecans 1–4 (Sdc 1–4), receptor tyrosine phosphatase-*β* (RPTP-*β*), anaplastic lymphoma kinase (ALK), integrins (*α*4*β*1 and *α*6*β*1 for MK and *α*v*β*3 for PTN) and low-density lipoprotein receptor-related protein [1, 17]. We have also demonstrated that the cell surface-nucleolin is a low-affinity receptor for both MK and PTN [15, 16]. Upon binding to their receptors, MK and PTN have some similarities in the signaling pathways that they induce. For instance, both cytokines can induce signaling through extracellular signal-regulated kinase (ERK1/2), phosphatidyl inositol 3-kinase/AKT, mitogen-activated protein kinase, and NOTCH-hairy and enhancer of split 1 (Hes-1) [5].

MK and PTN are produced in several types of cells and tissues. In adults, the significant expression of MK and PTN is detected only in restricted sites. For instance, MK is detected in the kidney [18], gut [19], epidermis [20], bronchial epithelium [21], and *B*-lymphocyte [22], and PTN is detected in the central nervous system, bladder, testis, the breast epithelium, stomach, and placental tissue [1]. Their expression is also detected at high levels in different types of tumors [2]. Moreover, MK and PTN were shown to be expressed by some types of immune cells. While MK was shown to be produced by T- and B cells, neutrophils, and mast cells [14, 23, 24], PTN production by these cells was not documented. In contrast, PTN was shown to be produced by some NK subtypes [25]. However, the production of MK by these cells was not reported. Moreover, MK production was detected in human monocytes and neutrophils [26], and its production by human macrophage-like cells differentiated from human monocytic cell line (THP-1) cells is debatable [27, 28]. We have recently demonstrated that MK is produced by human macrophages, monocyte-derived dendritic cells (MDDCs), and plasmacytoid dendritic cells (pDCs) upon stimulation through TLR4 [24]. In contrast, PTN production in human monocytes was not previously reported, and THP-1 does not have the capacity to produce PTN [29], although such production was detected in monocytic cells in quail [30]. However, PTN is produced by human tumor-associated macrophages [9]. To our knowledge, no study reported on the production of PTN by any type of dendritic cells (DCs). The production of MK and PTN by innate antigen-presenting cells (iAPCs) is an interesting aspect that requires further investigation. This is due to the fact that monocytes, macrophages, and DCs are implicated in many important physiological mechanisms, including tissue repair and angiogenesis [24, 31, 32], and that MK and PTN are also implicated in these processes [24, 33]. Interestingly, Toll-like receptors (TLRs) play an important role in tissue repair, angiogenesis, and immune responses against tumors and infections [34], especially TLRs 3, 7, 8, and 9, which can detect nucleic acids released from damaged and infected cells [35, 36].

In this study, we investigated whether the nucleic-acid sensing (NAS) TLRs can control MK and PTN production by innate APCs by assessing the production of these cytokines by monocytes, macrophages, MDDCs, mDCs, and pDCs upon TLRs 3, 7/8, and 9 triggering.

## 2. Materials and Methods

### 2.1. Study Population

Blood was obtained from 17 healthy donors (F/M ≈ 1, age = 29.8 ± 8 years). The number of donors in each experiment is indicated in the figures. Informed consent was obtained from all participants. The study was approved by the Medical Research Ethics Committee of the College of Medicine and Health Sciences in the Sultan Qaboos University (SQU) MERC#1654. The data analysis was done anonymously. For confidentiality, every donor was assigned with an identification number.

### 2.2. Isolation of Peripheral Blood Mononuclear Cells (PBMCs)

Blood samples were collected in CPDA-1 bags (Terumo, Japan). PBMCs were isolated from blood samples using the Ficoll-Hypaque density gradient technique (Sigma-Aldrich, Germany).

### 2.3. Isolation of Monocytes and Differentiation of Macrophages and MDDCs

Monocytes were isolated using a Pan Monocyte Isolation kit (Miltenyi Biotec, Germany) according to the manufacturer's instructions or by adherence for 2 hr in RPMI media containing no serum. Macrophages were differentiated from monocytes cultured under adherence conditions for 6 days in RPMI media (Gibco, UK) supplemented with 50 ng/ml human recombinant granulocyte-macrophage colony-stimulating factor (GM-CSF; R&D, UK). MDDCs were obtained from monocytes cultured under adherence conditions for 6 days in RPMI media supplemented with 50 ng/ml of human recombinant interleukin-4 (IL-4; R&D, UK) and 50 ng/ml GM-CSF.

### 2.4. Isolation of Myeloid DCs (mDCs) and pDCs

mDCs and pDCs were isolated from PBMCs by Myeloid Dendritic Cell Isolation kit, CD1c (BDCA-1)^+^ Dendritic Cell Isolation kit, and Plasmacytoid Dendritic Cell Isolation kit II (Miltenyi Biotech, Germany) according to the manufacturer's instructions.

### 2.5. Cell Culture

Monocytes, macrophages, MDDCs, and mDCs were stimulated at a concentration of 10^6^ cells/ml for 24 hr using 20 ng/ml lipopolysaccharide (LPS; InvivoGen, USA), poly I:C (20 ng/ml; Sigma-Aldrich, USA), resiquimod (5 *μ*g/ml; InvivoGen, USA) and CpG DNA (2 *μ*M; InvivoGen, USA). PDCs (at the concentration of 10^6^ cells/ml) were stimulated using resiquimod and CpG DNA at the same concentrations mentioned above (InvivoGen, USA) because they only have TLRs 7 and 9. Nonstimulated cells cultured under the same conditions were used as control. DCs were incubated for 24 hr. Cell count was determined before seeding using a hemacytometer, and the same number of cells was cultured in each well for all types of cells.

### 2.6. Flow Cytometry

Cell purity and activation (CD80 and CD86 upregulation) were assessed by flow cytometry LSRFortessa™ cell analyzer (BD, USA) using mouse monoclonal anti-human Abs anti-CD14 (557923, ID AB_396944), CD3 (561805, ID AB_10893800), CD56 (557919, ID AB_396940), CD19 (557921, ID AB_396942)-Alexa700, HLA-DR-APC-Cy7 (335831, ID AB_2868692), CD123-PE (340545, ID AB_400052), CD11c-APC (559877, ID AB_398680), CD80-PEcy5 (559370, ID AB_397239), and CD86-PEcy7 (561128, ID AB_10563077). The used concentrations were according to the manufacturer's instructions (BD, USA). The macrophages were identified as CD14^+^ cells and MDDCs as CD14^−^ cells.

### 2.7. Assessing MK and PTN Protein Production

The MK and PTN proteins were detected in the supernatant using the MK human ELISA kit (Biovendor, Germany) and PTN human ELISA kit (Aviva Systems Biology, USA) according to the manufacturer's instructions. The sensitivity of the PTN ELISA is <11.7 pg/ml, and the intra-assay coefficient of variation (CV) <10%. The sensitivity of the MK ELISA is <33 pg/ml, and the intra-assay CV < 5%. Values were considered positive if the replicates were significantly higher than the blank; otherwise, the production was considered nonsignificant or null. Semi-log standard curves were used, and values of the standard curve for PTN ranged from 31.2 to 2,000 pg/ml and for MK from 10 to 2,000 pg/ml.

### 2.8. Statistical Analysis

The significance of the observed differences between the two variables upon cell treatment was assessed using a 2-tailed paired and unpaired *t*-test. A *p*-value < 0.05 was considered significant. The error bars in the figures indicate the standard deviation. Microsoft Excel was used for statistical analysis.

## 3. Results

### 3.1. The Profile of the Obtained iAPCs

**Figure 1:**
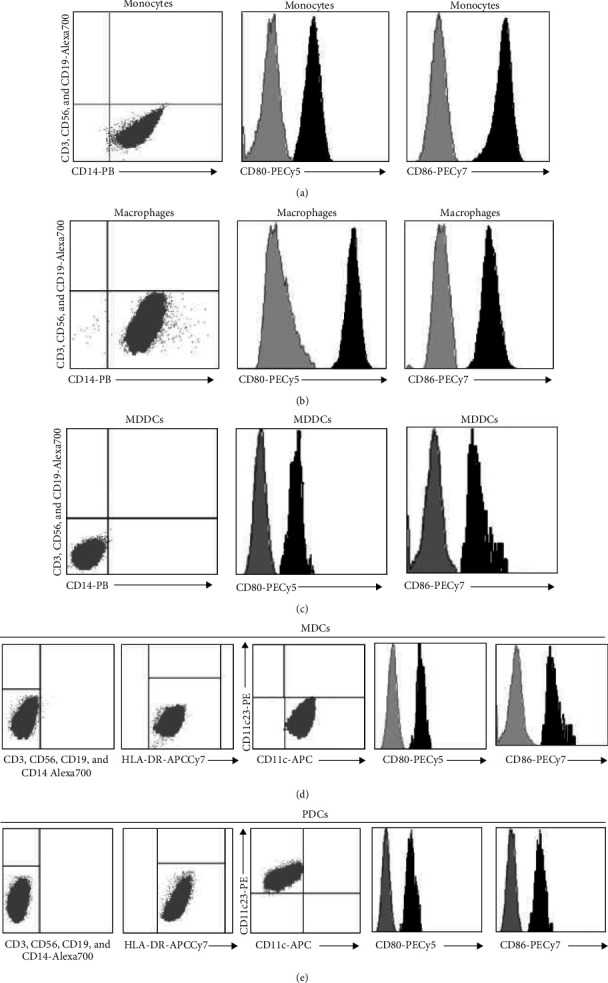
The profile of the iAPCs. Representative figures (plots and histograms) upon analysis by flow cytometry of (a) monocytes, (b) macrophages, (c) MDDCs, (d) mDCs, and (e) pDCs. The histograms illustrate the levels of CD80 and CD86 before (gray) and after (black) stimulation.

The obtained cells were analyzed by flow cytometry. The isolated monocytes (Figure 1(a)) and monocyte-derived macrophages (Figure 1(b)) were CD3^−^, CD56^−^, CD19^−^, and CD14^+^ and they upregulated CD80 and CD86 upon stimulation (Figures 1(a) and 1(b)). MDDCs were CD3^−^, CD56^−^, CD19^−^, and CD14^−^ and upregulated CD80 and CD86 upon stimulation (Figure 1(c)). Isolated mDCs were CD3^−^, CD56^−^, CD19^−^, CD14^−^, CD123^−^, HLA-DR^+^, and CD11c^+^ and they upregulated CD80 and CD86 upon stimulation (Figure 1(d)). Isolated pDCs were CD3^−^, CD56^−^, CD19^−^, CD14^−^, CD11^−^, HLA-DR^+^, and CD123^+^ and they upregulated CD80 and CD86 upon stimulation (Figure 1(e)).

### 3.2. The Production of MK and PTN in Primary Human Monocytes

We investigated whether both MK and PTN are produced by human monocytes upon stimulation through NAS TLRs. For this purpose, monocytes isolated from the blood were stimulated or not with poly I:C (20 ng/ml), a ligand of TLR3 that detects double-stranded RNA, resiquimod (5 *μ*g/ml), a ligand of TLR7/8 that detects single-stranded RNA and CpG DNA (2 *μ*M) the ligand of TLR9. As a control, LPS (20 ng/ml) was used to stimulate the cells.

**Figure 2:**
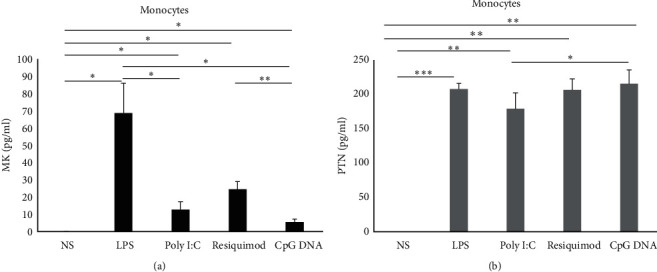
Production of MK and PTN by monocytes. Cells (*n* = 3) were stimulated or not with LPS, poly I:C, resiquimod, and CpG DNA. MK (a) and PTN (b) protein levels were measured in the supernatant by ELISA. The error bars indicate the standard deviation. NS, nonstimulated.

Monocytes produced MK when stimulated with LPS (average of 68.6 pg/ml, *p*-value = 0.02; Figure 2(a)), poly I:C (average of 12.7 pg/ml, *p*=0.043; Figure 2(a)), resiquimod (average of 24.5 pg/ml, *p*-value = 0.011; Figure 2(a)) and CpG DNA (average of 5.5 pg/ml, *p*-value 0.033; Figure 2(a)). In addition, monocytes produced high levels of PTN upon stimulation with LPS (average of 207.7 pg/ml, *p*-value = 0.0005; Figure 2(b)), poly I:C (average of 179.3 pg/ml, *p*=0.005; Figure 2(b)), resiquimod (average of 206.3 pg/ml, *p*-value = 0.002; Figure 2(b)) and CpG DNA (average of 215.3 pg/ml, *p*-value 0.0029; Figure 2(b)).

Interestingly, monocytes produced more PTN than MK when stimulated with LPS (*p*=0.008), poly I:C (*p*=0.009), resiquimod (*p*=0.002) and CpG DNA (*p*=0.003). Moreover, the levels of MK were significantly higher when stimulated with LPS compared to poly I:C (*p*=0.039; Table 1 and Figure 2(a)) and CpG DNA (*p*=0.026; Table 1 and Figure 2(a)) with a trend to be higher compared to resiquimod (*p*=0.055; Table 1). The levels of MK produced were also significantly higher when stimulated by resiquimod compared to CpG DNA (*p*=0.007; Table 1 and Figure 2(a)). In contrast, PTN levels were significantly higher when cells were stimulated using CpG DNA compared to poly I:C (*p*=0.01; Table 1 and Figure 2(a)).

The CV (%) for MK ELISA replicates varied from 1.1% to 11.1%, and that for PTN ELISA replicates from 0.9% to 5.9%.

### 3.3. A Differential Production of MK and PTN by Macrophages

**Figure 3:**
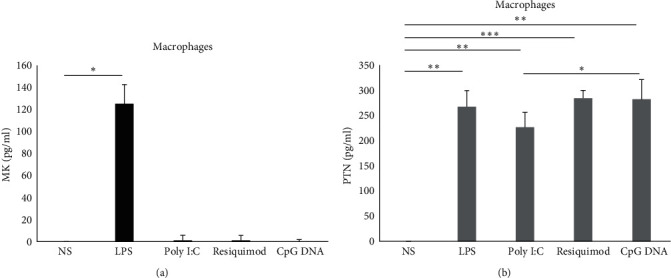
Production of MK and PTN by macrophages. Cells (*n* = 3) were stimulated or not with LPS, poly I:C, resiquimod, and CpG DNA. MK (a) and PTN (b) protein levels were measured in the supernatant by ELISA. The error bars indicate the standard deviation. NS, nonstimulated.

Macrophages were stimulated under the same conditions mentioned above. They significantly produced MK when stimulated with LPS (average of 125 pg/ml, *p*=0.016; Figure 3(a)). However, a nonsignificant, very low level of MK was produced when cells were stimulated with poly I:C and resiquimod (Figure 3(a)), and no production was observed upon stimulation with CpG DNA (Tables 1 and 2). In contrast, macrophages produced PTN under all conditions LPS (average of 267.7 pg/ml, *p*-value = 0.0046; Figure 3(b)), poly I:C (average of 226.7 pg/ml, *p*=0.0057; Figure 3(b)), resiquimod (average of 284.7 pg/ml, *p*-value = 0.00095; Figure 3(b)) and CpG DNA (average of 282.3 pg/ml, *p*-value 0.0064; Figure 3(b)).

Macrophages produced more PTN than MK when stimulated with poly I:C (*p*=0.007), resiquimod (*p*=0.001), and CpG DNA (*p*=0.006), and a trend of a higher production of PTN was also observed with LPS (*p*=0.056). Similar to what was observed in monocytes, PTN levels were significantly higher when cells were stimulated using CpG DNA compared to poly I:C (*p*=0.02; Table 1 and Figure 3(b)).

The CV (%) for MK ELISA replicates varied from 0.8% to 4.4%, and that for PTN ELISA replicates from 0.6% to 7.5%.

### 3.4. The Production of MK and PTN by MDDCs

**Figure 4:**
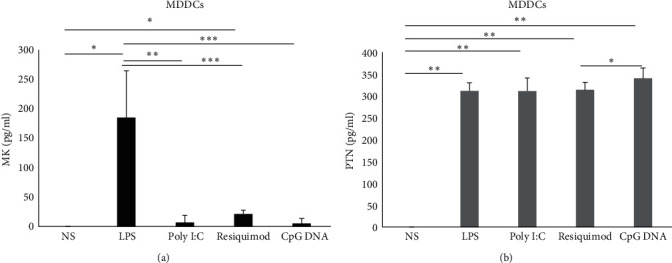
Production of MK and PTN by MDDCs. Cells (*n* = 3) were stimulated or not with LPS, poly I:C, resiquimod, and CpG DNA. MK (a) and PTN (b) protein levels were measured in the supernatant by ELISA. The error bars indicate the standard deviation. NS, nonstimulated.

MDDCs were stimulated under the same conditions mentioned above. MDDCs produced significant amounts of MK when stimulated with LPS (average of 184.8 pg/ml, *p*=0.01; Figure 4(a)) and resiquimod (average of 21.3 pg/ml, *p*=0.026; Figure 4(a)). However, a nonsignificant low level of MK was observed when cells were stimulated with poly I:C and CpG DNA (Figure 4(a)). In contrast, MDDCs produced PTN under all conditions LPS (average of 311.6 pg/ml, *p*-value = 0.0012; Figure 4(b)), poly I:C (average of 311 pg/ml, *p*=0.003; Figure 4(b)), resiquimod (average of 313.7 pg/ml, *p*-value = 0.001; Figure 4(b)) and CpG DNA (average of 340 pg/ml, *p*-value 0.0016; Figure 4(b)).

Similar to macrophages, MDDCs produced more PTN than MK when stimulated with poly I:C (*p*=0.001), resiquimod (*p*=0.002), and CpG DNA (*p*=0.003). Moreover, MDDCs produced significantly higher levels of MK when stimulated with LPS compared to poly I:C (*p*=0.001; Table 1 and Figure 4(a)), resiquimod (*p*=0.0003; Table 1 and Figure 4(a)) and CpG DNA (*p*=0.0004; Table 2). PTN levels were significantly higher when cells were stimulated with CpG DNA compared to resiquimod (*p*=0.03; Table 1 and Figure 4(a)).

The CV (%) for MK ELISA replicates varied from 1.9% to 7.5%, and that for PTN ELISA replicates from 0.3% to 6.1%.

### 3.5. MDCs Produce PTN Only

**Figure 5:**
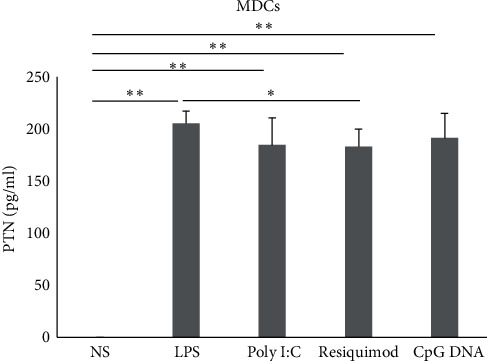
Production of PTN by mDCs. Cells (*n* = 3) were stimulated or not with LPS, poly I:C, resiquimod, and CpG DNA. PTN protein levels were measured in the supernatant by ELISA. The error bars indicate the standard deviation. NS, nonstimulated.

MDCs did not produce MK under any stimulation condition, regardless of the isolation method described above. In contrast, MDCs produced PTN under all the stimulation conditions LPS (an average of 205.3 pg/ml, *p*=0.0011; Figure 5), poly I:C (an average of 184.7 pg/ml, *p*=0.006; Figure 5), resiquimod (an average of 183 pg/ml; *p*=0.0028; Figure 5) and CpG DNA (an average of 191.3 pg/ml, *p*=0.005; Figure 5). MDCs produced significantly higher levels of PTN when stimulated with LPS compared to resiquimod (*p*=0.07; Table 1 and Figure 5).

The CV (%) for PTN ELISA replicates varied from 1% to 10.7%.

### 3.6. The Production of MK and PTN by pDCs

**Figure 6:**
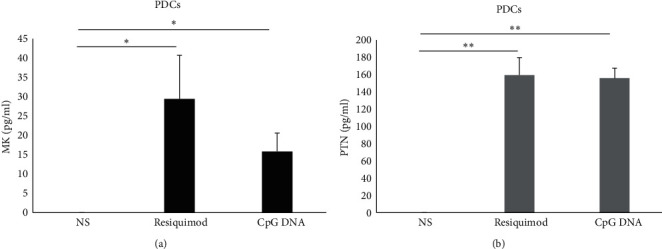
Production of MK and PTN by pDCs. Cells (*n* = 3) were stimulated or not with resiquimod and CpG DNA. MK (a) and PTN (b) protein levels were measured in the supernatant by ELISA. The error bars indicate the standard deviation. NS, nonstimulated.

PDCs were stimulated with resiquimod and CpG DNA because these cells have TLRs7 and 9 only. PDCs produced MK when stimulated with resiquimod (an average of 29.4 pg/ml, *p*=0.045; Figure 6(a)) and CpG DNA (an average of 15.8 pg/ml, *p*=0.028; Figure 6(a)). Moreover, pDCs produced PTN when stimulated with resiquimod (an average of 159.3 pg/ml, *p*=0.0054; Figure 6(b)) and CpG DNA (an average of 155.7 pg/ml, *p*=0.0018; Figure 6(b)).

Similar to the other investigated APCs, pDCs produced significantly higher levels of PTN compared to MK when stimulated with resiquimod (*p*=0.003) and CpG DNA (*p*=0.004).

The CV (%) for MK ELISA replicates varied from 4.4% to 16.5%, and that for PTN ELISA replicates from 5.3% to 4.1%.

### 3.7. Differences in the Capacity of Innate APCs to Produce MK

We compared the innate APCs in their capacity to produce MK upon stimulation through the different NAS TLRs. As mentioned above, mDCs did not produce MK under any simulation condition; therefore, the levels of their production cannot compare to those in cells that produced significant levels of MK (Table 2). When comparing the levels of MK between the other innate APCs, we observed that monocytes produced significantly higher levels of MK compared to macrophages when stimulated with poly I:C (*p*=0.017; Table 2), resiquimod (*p*=0.001; Table 2), and CpG DNA (*p*=0.006; Table 2). Moreover, pDCs and MDDCs produced higher levels of MK compared to macrophages upon stimulation with resiquimod (*p*=0.013 and 0.005, respectively; Table 2). Moreover, pDCs produced higher levels of MK compared to macrophages and monocytes upon stimulation with CpG DNA (*p*=0.004 and 0.024, respectively; Table 2).

### 3.8. Differences in the Capacity of Innate APCs to Produce PTN

We also compared the innate APCs in their capacity to produce PTN upon stimulation through the different NAS TLRs. We observed that MDDCs produced significantly higher levels of PTN compared to monocytes (*p*=0.0009; Table 2) and mDCs (*p*=0.001; Table 2) when stimulated with LPS. They also produced significantly higher levels of PTN compared to monocytes (*p*=0.004; Table 2) and mDCs (*p*=0.005; Table 2) when stimulated with poly I:C. Similarly, macrophages produced significantly higher levels of PTN compared to monocytes and mDCs (*p*=0.034 and 0.03, respectively; Table 2) when stimulated with LPS.

A similar pattern was observed when cells were stimulated with resiquimod, where MDDCs produced significantly higher levels of PTN compared to monocytes (*p*=0.001; Table 2), mDCs (*p*=0.0007; Table 2), and pDCs (*p*=0.0006; Table 2). Macrophages also produced significantly higher levels of PTN compared to monocytes (*p*=0.003; Table 2), mDCs (*p*=0.001; Table 2), and pDCs (*p*=0.001; Table 2) when stimulated with resiquimod.

In addition, MDDCs produced the highest levels of PTN when compared to monocytes (*p*=0.001 0.002; Table 2), mDCs (*p*=0.002; Table 2) and pDCs (*p*=0.0002; Table 2), when stimulated with CpG DNA. Macrophages also produced significantly higher levels of PTN compared to monocytes (*p*=0.047; Table 2), mDCs (*p*=0.026; Table 2) and pDCs (*p*=0.006; Table 2), when stimulated with CpG DNA. Moreover, monocytes produced significantly higher levels of PTN compared to pDCs (*p*=0.035 and 0.01 resiquimod and CpG DNA, respectively; Table 2).

## 4. Discussion

Our results demonstrates for the first time that PTN is produced by primary human monocytes and their derived DCs in addition to mDCs and pDCs. PTN production was observed upon triggering through the NAS TLRs 3, 7/8, and 9 and TLR4. Our study confirms the capacity of macrophages to produce PTN and demonstrate for the first time that this production can be controlled by TLR triggering, including the triggering through the NAS TLRs.

In contrast, MK production was not observed under all conditions in the investigated innate APCs. While monocytes and pDCs produced MK upon triggering through all their NAS TLRs, macrophages significantly produced MK only upon triggering through TLR4. However, TLRs 3 and 7/8 triggering induced a slight production of MK and TLR 9 triggering did not induce MK production, indicating a weak to no response in terms of MK production upon NAS TLR triggering in macrophages. Moreover, MDDCs significantly produced MK only upon triggering through TLRs 4 and 7/8, while TLRs 3 and 9 triggering induced a slight production of MK. This indicates a differential capacity to induce MK production among NAS TLRs. These results demonstrated that the same TLRs can induce different immune responses in the different innate APCs. This difference in the capacity of innate APCs to produce cytokines was also described by us and others for cytokines, including IFNs [37, 38]. This can be due to the activation of distinct pathways and transcription programs that can control the production of the cytokines in the different APCs. For instance, our results demonstrating the production of MK and PTN by monocytes differ from those reported with THP-1 cells in which MK production is debatable [27, 28] and which do not have the capacity to produce PTN [29]. This supports the hypothesis that the different pathways and transcription programs that are present in the cells, although these cells might have similarities like monocytes and THP-1 cells, can influence MK and PTN production. This confers unique yet complementary and overlapping capacities to each type of APCs.

We also report for the first time that innate APCs produced higher levels of PTN compared to MK under all conditions. This is in line with the fact that the magnitude of production differs among cytokines in innate APCs [38, 39]. MK and PTN genes are located on different chromosomes in human, 11q11.2 for *MDK* [2], and *PTN* is located on the 7q33 chromosome [4]. The regulation of both cytokines is also different [1]. We and others have shown that NF-*κ*B can regulate MK production, including the production upon TLR triggering in innate APCs [24]. However, transcription factors like AP1 can regulate the production of PTN [4]. Moreover, MK production itself might regulate the production of PTN, and an elevated production of PTN was observed in the absence of MK, suggesting a compensatory role for PTN [40]. These differences provide rationale for the observed differences in the production of MK and PTN.

Moreover, triggering through TLR4 had a higher capacity to induce MK production in monocytes compared to triggering through TLRs 3 and 9 and a tendency to be higher than TLRs7/8. This was also observed in MDDCs, where triggering through TLR4 induced higher levels of MK compared to triggering through TLRs 3, 7/8, and 9. This is consistent with the fact that triggering through TLR4 was the only condition under which macrophages produced significant levels of MK. This indicates that TLR4 has a stronger capacity to induce MK production compared to NAS TLRs in monocytes and their derived macrophages and DCs. LPS that triggers TLR4 is a strong inducer of cytokines in innate APCs [41]. This distinct effect of LPS can be due to the different pathways that TLR4 uses to induce an immune response, including the fact that it induces both MyD88 and TRIF pathways, in comparison with the other TLRs that induce either TRIF in the case of TLR3 or MyD88 in the case of the other TLRs, including TLRs 7/8 and 9 [36]. In contrast, PTN production was at comparable levels under the majority of conditions, with a slightly significant difference between the triggering through TLRs3 and 9 in monocytes and macrophages and between TLRs 7/8 and 9 in MDDCs. TLRs differ in their capacity to induce the different cytokines in the innate APCs [36]. This is potentially regulated by the various pathways that they might induce and the different factors that can control them in the innate APCs. Human iAPCs express TLRs 1–10 with the exception of pDCs that express TLRs 7 and 9 only [42, 43]. The latter express high levels of TLRs 7 and 9, while unstimulated monocytes have high levels of all expressed TLRs except TLR7 [44]. The level of TLRs might decrease in these cells when they differentiate into macrophages and MDDCs [45], although their capacity to produce cytokines increases upon differentiation [46], which might result from differences in the signaling pathways. These differences in TLR levels and the potency of their signaling pathways between iPACs might provide rational for the differences in the levels of MK and PTN produced by iAPCs upon TLR triggering. In general, monocytes produced higher levels of PTN after they differentiated into macrophages and MDDCs. MDDCs produced the highest levels of PTN compared to the other innate APCs. The differentiation of monocytes increases their capacity to produce some cytokines [46]. This might be the result of the differentiation-induced divergence in the pathways that can control the induction of the cytokines.

The differences in MK and PTN production that were observed between the different conditions in our model are not due to differences in the number of seeded cells. This is supported by the fact that the same number of cells was cultured in each well for all types of cells. In addition, the experiments were repeated with different donors, and the significance in the difference reflects a consistency in the results that is unlikely to occur randomly. Moreover, MK and PTN were measured in the same supernatant; therefore, if the differences in the production, whether within the same type of cytokine or between the two cytokines, were due to differences in the cell density, this would have had the same effect on both cytokines in the same time, which is not the case, e.g., the same cells produce higher levels of PTN than MK, MK levels are significantly different between two conditions (cell type, ligand) but not PTN and vice versa.

MK and PTN play an important role in angiogenesis [1, 15, 24, 30]. It is interesting to investigate how the innate APCs can modulate angiogenesis and the proliferation of endothelial cells through the differential production of MK and PTN under the different inflammatory conditions, especially under the conditions of tumors and tissue damage where the NAS TLRs have a major role in detecting the damage associated molecular patterns resulting for the injured cells [47].

Moreover, a number of studies demonstrated that MK and PTN are implicated in the modulation of the immune responses [1]. Innate APCs, especially macrophages and DCs, have a major influence on the capacity of the cells of the adaptive immune system to survive and mount responses. Macrophages and DCs can increase the survival of B cells [48, 49]. MK and PTN can induce signaling cascades that result in the survival of B cells [1, 22]. This highlights the importance of assessing whether MK and PTN have a role in the capability of innate APCs to regulate B-cell survival. MK and PTN can also affect T cells [1]. PTN can induce the production of inflammatory responses in PBMCs [1]. In addition, MK can activate CD4 T cells and play a role in the differentiation of T helper 1 (Th1) cells [50]. It also has an inhibitory effect on the differentiation of regulatory T cells (T Regs) [1, 24]. In contrast, MK effects on CD8 T cells require more investigations. While a study showed an activating effect on CD8 T cells, another study described an indirect ability to promote CD8 T-cell dysfunction [51, 52]. Knowing the important role of innate APCs in the activation of T cells [53, 54], it is very important to investigate the role of both MK and PTN produced by innate APCs in the capacity of these cells to control T-cell responses. The differential and coproduction of MK and PTN by innate APCs can have different impacts on T and B cells under the conditions of tumors and infections, where the NAS TLRs have a major role in detecting the damage and pathogen-associated molecular patterns [36], particularly through TLRs 3, 7, 8, and 9 that do have different capacities in the induction of MK and PTN.

MK and PTN were also shown to be associated with autoimmune diseases such as RA [10, 11], MS [12, 13], cancers [2, 9], and the inhibition of HIV infection [14–16]. Therefore, assessing the production of MK and PTN by innate APCs in these situations and regulating their production by these cells is potentially important for the better management of these diseases.

**Figure 7:**
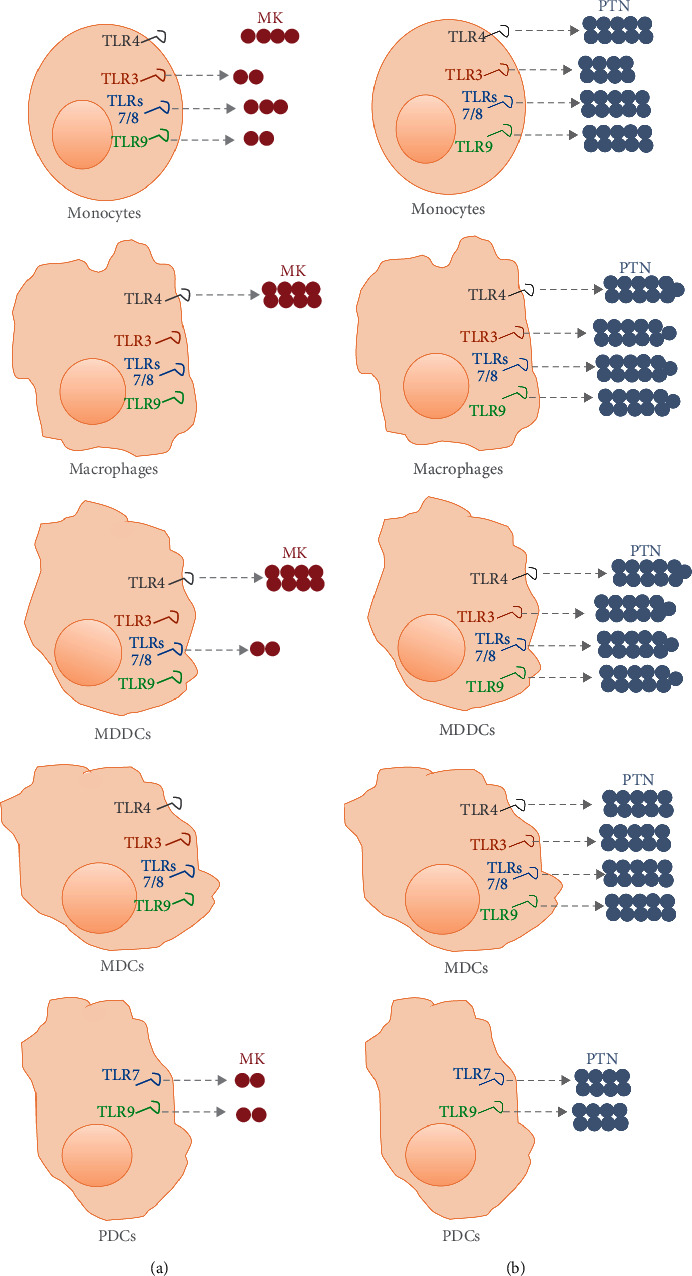
Schematic representation of the differential MK and PTN production by iPACs upon TLR triggering. (a) The production of MK by iAPCs. (b) The production of PTN by iAPCs. IAPCs produce higher levels of PTN than MK upon triggering through TLR4 and NAS TLRs. All NAS TLR can induce PTN production in iAPCs, and monocytes produce higher levels of PTN when they differentiate into macrophages and MDDCs. MDCs do not produce MK upon TLR triggering. NAS TLRs can induce MK production by monocytes and pDCs; however, only TLRs7/8 among them induce MK production by MDDCs, and none of them induce MK production by macrophages. In monocytes, macrophages, and MDDCs TLR4 induces a higher level of MK production compared to NAS TLRs.

Altogether, our results demonstrated that MK and PTN are produced by innate APCs upon their stimulation through the NAS TLRs. However, the production of these cytokines differs among these APCs (Figures 7(a) and 7(b)), with a more pronounced production of PTN compared to MK and a selective production of MK in the different innate APCs depending on the triggered NAS TLR.

## Figures and Tables

**Table 1:** Comparison of different stimuli effect on MK and PTN production in the same cells.

Cytokine	LPS	Poly I:C	Resiquimod	CpG DNA	*p*-Value					
					L P	L R	L C	P R	P C	R C
Monocytes										
MK	68.6	12.7	24.5	5.5	0.039	0.055	0.026	0.15	0.19	0.007
PTN	207.7	179.3	206.3	215.3	0.09	0.86	0.40	0.18	0.01	0.49
Macrophages										
MK	125.2	1.1	1.1	0	0.17	0.17	0.16	0.42	0.42	0.42
PTN	267.7	226.7	284.7	282.3	0.08	0.33	0.23	0.09	0.02	0.92
MDDCs										
MK	184.8	6.8	21.3	4.9	0.001	0.0003	0.0004	0.059	0.870	0.130
PTN	311.7	311	313.7	340	0.98	0.44	0.04	0.92	0.42	0.03
MDCs										
MK	0	0	0	0	–	–	–	–	–	–
PTN	205.3	184.7	183.0	191.3	0.21	0.07	0.25	0.93	0.20	0.62
PDCs										
MK	–	–	29.4	15.8	–	–	–	–	–	0.11
PTN	–	–	159.3	155.7	–	–	–	–	–	0.86

L P: LPS—Poly I:C, L R: LPS—resiquimod, L C: LPS—CpG DNA, P R: Poly I:C—resquimod, P C: Poly I:C—CpG DNA, R C: resiquimod CpG DNA and the standard deviation (S.D.) values for the average levels in this table are the same in Table 1.

**Table 2:** Comparison of the same stimulus effect on MK and PTN production between different cells.

Cytokine	Monocytes	Macrophages	MDDCs	MDCs	PDCs	*p*-Value									
						Mo/Ma	Mo/MDDC	Mo/MDC	Mo/PDC	Ma/MDDC	Ma/MDC	Ma/PDC	MDDC/MDC	MDDC/PDC	MDC/PDC
LPS															
MK	68.6	125.2	184.8	0	–	0.48	0.29	0.021	–	0.57	0.004	–	0.14	–	–
PTN	207.7	267.7	311.7	205.3	–	0.034	0.0009	0.8389	–	0.1	0.03	–	0.001	–	–
Poly I:C															
MK	12.7	1.1	6.8	0	–	0.017	0.55	0.0434	–	0.52	0.4	–	0.42	–	–
PTN	179.3	226.7	311	184.7	–	0.03	0.004	0.6875	–	0.071	0.007	–	0.005	–	–
Resquimod															
MK	24.5	1.1	21.3	0	29.4	0.001	0.61	0.0115	0.6	0.005	0.4	0.013	0.026	0.96	0.046
PTN	206.3	284.7	313.7	183.0	159.3	0.004	0.001	0.3354	0.035	0.062	0.001	0.001	0.0007	0.0006	0.2
CpG DNA															
MK	5.5	0	4.9	0	15.8	0.006	0.930	0.034	0.024	0.42	–	0.004	0.42	0.2	0.029
PTN	215.3	282.3	340	191.3	155.7	0.047	0.002	0.2539	0.01	0.09	0.026	0.006	0.002	0.0002	0.151

Mo, monocytes and Ma, macrophages. The standard deviation (S.D.) values for the average levels in this table are the same in Table 1.

## Data Availability

All relevant data are available in the manuscript.
